# Trimetazidine Inhibits Renal Tubular Epithelial Cells to Mesenchymal Transition in Diabetic Rats *via* Upregulation of Sirt1

**DOI:** 10.3389/fphar.2020.01136

**Published:** 2020-07-29

**Authors:** Yong Yang, Yong Wang, Zuowen He, Yunchang Liu, Chen Chen, Yan Wang, Dao Wen Wang, Hong Wang

**Affiliations:** ^1^ Division of Cardiology, Department of Internal Medicine, Tongji Hospital, Tongji Medical College, Huazhong University of Science and Technology, Wuhan, China; ^2^ Hubei Key Laboratory of Genetics and Molecular Mechanisms of Cardiologic Disorders, Huazhong University of Science and Technology, Wuhan, China

**Keywords:** diabetic nephropathy, epithelial-to-mesenchymal transition, trimetazidine, Sirt1, reactive oxygen species

## Abstract

Trimetazidine (TMZ), as a metabolic regulator, is effective in treatment of coronary atherosclerotic heart disease with rare side effects in the clinic for long years. Interestingly, studies have shown that TMZ protects against several acute kidney injuries (AKI). However, the effect of TMZ on chronic kidney diseases (CKD) remains unknown. This study aimed to investigate the role of TMZ in diabetic nephropathy (DN) and its potential mechanisms. A rat model of DN was established in male Sprague-Dawley rats by streptozotocin (STZ) intraperitoneal injection. Experimental rats were separated into three groups: control, DN and DN + TMZ treatment. Metabolic parameters, pathological features and renal function markers were evaluated after 20 weeks of diabetes induction. *In vitro* experiments, the effect of TMZ on high fat and high glucose (HFG) induced or TGFβ1-induced epithelial-to-mesenchymal transition (EMT) was examined in HK-2 cells. Our results showed that TMZ could maintain renal function without affecting hemodynamic and plasma metabolic levels in diabetic rats. The effect was associated with a reversion of pathological progression of DN, especially for tubulointerstitial fibrosis. EMT is an important contributor to renal fibrosis. In this study, we investigated the role of TMZ in the process of EMT in DN. Mechanistically; TMZ attenuated HFG-induced EMT by relieving oxidative stress *via* deacetylation forkhead box O1 (FoxO1) in a Sirt1-dependent pathway. And it suppressed TGFβ1-induced EMT by deacetylating Smd4 in a Sirt1-dependent manner. Moreover, our study found that TMZ upregulated Sirt1 expression by increasing the expression of nicotinamide phosphoribosyl transferase (Nampt), which is a rate limiting enzyme for nicotinamide adenine dinucleotide (NAD^+^) generation by salvage pathway. And the increased NAD^+^ promoted Sirt1 expression. In conclusion, TMZ can prevent renal dysfunction and pathogenesis of tubulointerstitial fibrosis in DN, partly by inhibition of EMT *via* FoxO1/ROS pathway and TGFβ/Smad pathway in a Nampt/NAD^+^/Sirt1 dependent manner.

## Introduction

Diabetic nephropathy (DN) is the leading cause of end-stage renal disease and increases the risk of death (Tuttle et al., 2014; Webster et al., 2017; Li et al., 2019). Glomerular lesions were previously considered the primary cause of renal function decline in the process of DN. However, the kidney is mainly composed of renal tubules, and the renal tubules are more sensitive to the injury of metabolic disorders than glomeruli (Gilbert, 2017). Emerging evidence suggests that renal tubular lesions play an important role in the occurrence and progression of this disease (Vaidya et al., 2011; Gilbert, 2017; Zeni et al., 2017). For example, tubulointerstitial fibrosis, which is the common final outcome of tubular lesions, is the most important predictor of the progression of DN (Taft et al., 1994; Grgic et al., 2012; Okada et al., 2012).

Renal tubular epithelial cells (TECs) can convert to myofibroblasts *via* a process known as epithelial-to-mesenchymal transition (EMT), which is characterized by the loss of an epithelial marker and an increased mesenchymal phenotype, results in increased production of extracellular matrix (ECM) and contributes to the development of chronic kidney diseases (CKD) (Hay and Zuk, 1995). Studies have shown that renal fibrosis could be ameliorated by blunting EMT in a variety of kidney diseases (Yang and Liu, 2002; Grande et al., 2015). In diabetes, high fat and high glucose (HFG) led to elevated levels of reactive oxygen species (ROS), transforming growth factor-β1 (TGFβ1), and other factors, thereby inducing EMT in TECs (Ha and Lee, 2003; Hills and Squires, 2011; Chen et al., 2018). And inhibition of EMT could alleviate renal dysfunction and the progression of tubulointerstitial fibrosis in DN (Shi et al., 2017; Zhao et al., 2017).

Trimetazidine (TMZ), a piperazine derivative, has been widely used in the treatment of coronary atherosclerotic heart disease *via* shifting cardiac energy metabolism from fatty acid oxidation to glucose oxidation (Kantor et al., 2000; Ferrari et al., 2019). TMZ can also alleviate fibrosis in diabetic cardiomyopathy (Zhang et al., 2016) or inhibit cardiac fibrosis induced by pressure-overload (Liu et al., 2010). Other than the beneficial effects on the heart, studies have shown the protective effects of TMZ on acute kidney injuries (AKI), such as contrast nephropathy, drug-induced nephrotoxicity, and ischemia/reperfusion injury (Hauet et al., 2000; Satyanarayana and Chopra, 2002; Onbasili et al., 2007). However, whether TMZ has a positive effect on CKD, specifically in cases of renal fibrosis and EMT, is rarely studied.

Sirt1, a protein deacetylase, is an essential modulator of cellular energy metabolism and aging (Rahman and Islam, 2011; Catana et al., 2018; Vasquez and Tomanek, 2019). It has been shown to suppress oxidative stress in kidney injuries (Wu et al., 2012; Dong et al., 2014) and is capable of inhibiting multiple organ fibrosis (Simic et al., 2013). Previous article showed that TMZ had an anti-ROS effect in sepsis-induced myocardial dysfunction *via* the activation of Sirt1 (Chen et al., 2016). We suspected that TMZ may affect the expression level of Sirt1 in DN and had a protective effect. ROS pathway and TGFβ/Samd pathway play a crucial role in the process of organ fibrosis (Xu et al., 2009; Wu et al., 2012). Transcriptional factor FoxO1 is regulated by Sirt1 and plays a role in anti-oxidative stress in DN (Wu et al., 2012), and Sirt1 inhibits the TGFβ/Smad pathway by deacetylating Smad2/3/4/7 (Garcia-Vizcaino et al., 2017; Wang et al., 2018). We speculated that Sirt1/FoxO1/ROS pathway and Sirt1/TGFβ/Smad pathway may be involved in the regulation of DN by TMZ. In the current study, we confirmed that TMZ could ameliorate renal fibrosis by inhibiting EMT and prevent the progression of CKD in a STZ-induced diabetic rat model. To further study the underlying mechanism, we investigated the role of Sirt1 in the process of EMT and how TMZ affects Sirt1 expression. Sirt1/FoxO1/ROS pathway and Sirt1/TGFβ/Smad pathway were involved in the inhibitory effect of TMZ on EMT in DN. In addition, our findings revealed that TMZ upregulates Sirt1 expression through the Nampt/NAD^+^ pathway.

## Materials and Methods

### Reagents

Streptozotocin (STZ, V900890) was purchased from Sigma-Aldrich (St. Louis, MO). TMZ was provided by Servier (Tianjin China). Sirt1 siRNA and non-targeted siRNA were purchased from RiboBio (Guangzhou China). Lipofectamine™ 2000 Transfection Reagent (11668019) was from Invitrogen (Carlsbad, CA, USA). Antibodies against Sirt1 (A11267), FN (A12932), Sirt3 (A5718), and pan-acetyl lysine (A2391) were purchased from Abclonal (Cambridge, MA, USA). Antibodies against E-cadherin (20874-1-AP), Col1a1 (A1352), α-SMA (55135-1-AP), TGFβ1 (21898-1-AP), FoxO1 (18592-1-AP), Smad4 (10231-1-AP), Nampt (11776-1-AP), and β-actin (60008-1-Ig) were purchased from Proteintech Group (Wuhan, China). Antibody against TGFβRI (sc-518018) was from Santa Cruz Biotechnology (Dallas, TX, USA). Antibody against p-Ampk (50081) was from Cell Signaling Technology (Danvers, MA, USA). Dihydroethidium (DHE, S0063), N-acetyl-L-cysteine (NAC, S0077), hydrogen peroxide detection kit (S0038), Sod activity detection kit (S0101), and NAD^+^/NADH detection kit (S0175) were from Beyotime Biotechnology (Shanghai China). NAD^+^ (HY-B0445), FK866 (HY-50876), and Compound C (CC, HY-13418) were purchased from MedChemExpress (Shanghai China).

### Animals and Experimental Protocol

Male Sprague-Dawley rats (8 weeks old, 200–220 g) were purchased from Hubei Research Centre of Laboratory Animals (Wuhan, China). Diabetes was induced by intraperitoneal injection of STZ (55 mg/kg in 0.1 mol/L citrate buffer, adjusted to pH 4.5). Rats in the control group received equal volumes of citrate buffer (n = 7). Diabetes was verified 72 h later after STZ injection by measuring blood glucose levels. Rats with fasting blood glucose > 11.1 mmol/L were selected as qualified diabetic models and used in the study (n = 15). Two weeks after the onset of diabetes, diabetic rats were randomly divided into two groups: rats in the DN group (n = 8) received vehicle (0.9% saline solution), and rats in the DN + TMZ group (n = 7) received TMZ (5 mg/kg/day), respectively. The medications were given by oral gavage. After 20 weeks of treatment, blood and urine samples were taken for metabolite measurements. The rats were then sacrificed, and tissue samples were collected and stored at −80°C for paraffin embedding or snap frozen. All animal studies were approved by the Animal Research Committee of Tongji Medical College and followed the guidelines of the National Institutes of Health.

### Analysis of Plasma and Urine Metabolites

Plasma levels of glucose, triglyceride, cholesterol, creatinine, and urea nitrogen and urine levels of albumin, creatinine, and N-acetyl-β-D-glucosaminidase (β-NAG) were detected using assay kits from Nanjing Jiancheng Bioengineering Institute (Nanjing, China) following the manufacturer’s instructions.

### Histology and Immunohistochemical Staining

Kidney tissues were ﬁxed with 4% paraformaldehyde, then dehydrated and embedded in paraffin. Sections (4 mm thick) were subjected to hematoxylin-eosin (HE), periodic acid Schiff (PAS), and Sirius Red staining. Glomerular area, mesangial area/glomerular area, and proximal tubular inner diameter were measured as previously described (Gotoh et al., 2010; Wu et al., 2015; Irsik et al., 2018). Glomerular collagen content percentage was calculated by comparing the Sirius Red staining positive area in glomeruli to the total glomerular area. Tubulointerstitial collagen content percentage was calculated by comparing the Sirius Red staining positive area in the renal tubulointerstitium to the total renal tubular interstitial area. The ultrastructure of the kidney was observed using a transmission electron microscope (TEM; Hitachi, Tokyo, Japan), according to the method described previously (Kalimuthu et al., 2018). Immunohistochemistry and immunofluorescence staining were conducted as described previously (Oshimori and Fuchs, 2012; Wang et al., 2019).

### Cell Culture, Transfection, and Treatment

HK-2 cell lines were purchased from the Cell Bank of the Chinese Academy of Sciences (Shanghai, China), and grown in DMEM/F12 supplemented with 10% FBS. Cells were transfected with siRNA against human Sirt1 (si-Sirt1, 100 nM) or negative control (si-NC, 100 nM), using Lipofectamine 2000, according the manufacturer’s protocol. After transfection, cells were incubated with normal glucose (5 mM) or high fat (100 μM Palmitic acid) and high glucose (30 mM) for 48 h and then collected.

### Western Blotting

Kidney tissues and cells were collected using Protein or IP lysate (Beyotime Technology, Shanghai, China). The protein concentration was measured by BCA kit (Boster, Wuhan, China). A total of 10% gradient gels were used. The α-SMA antibody was diluted at 1:3,000, the β-actin antibody at 1:5,000, and the others at 1:1,000. Density analysis of the bands was performed using Gel Pro analysis software.

### Detection of Oxidative Stress Indexes

In order to reflect the degree of oxidative stress in tissues and cells under different treatment conditions, H_2_O_2_ levels, Sod activity, and DHE staining were all used for detection, according to the kit instructions.

### Protein Acetylation Assay

Forkhead box O1 (FoxO1) and Smad4 acetylation were measured by immunoprecipitation of collected protein lysate using antibody against FoxO1 and Smad4 respectively. Thereafter, acetylated-FoxO1 and acetylated-Smad4 were assessed with anti-pan acetyl lysine antibody.

### Detection of NAD^+^/NADH

The levels of nicotinamide adenine dinucleotide (NAD^+^) and NADH in kidney tissue and HK-2 cells were detected by the NAD^+^/NADH assay kit, according to the manufacturer’s instructions. In brief, renal cortex (10 mg/sample) and HK-2 cells (1×10^6^ cells/sample) were lysed with 200 μl of NAD^+^/NADH extraction reagent. In order to detect the total amount of NAD^+^ and NADH, 20 μl of lysate was added to a 96-well plate. To determine the content of NADH, the lysates was incubated at 60°C for 30 min to remove NAD^+^, and then 20 μl of supernatant was added to a 96-well plate. Subsequently, 90 μl of alcohol dehydrogenase working solution was added and incubated at 37°C for 10 min. Finally, 10 μl of color reagent was added to the 96-well plate and incubated at 37°C for 30 min. The absorbance was measured at 450 nm. According to the standard curve, the total content of NAD^+^/NADH and the content of NADH were calculated. The NAD^+^ content was equal to the total content minus the NADH content.

### Statistics

Results were expressed as mean ± SEM. One-way analysis of variance (ANOVA) with Newman-Keuls post analysis was used to compare the means of three or more different treatment groups. A *P* < 0.05 was considered to be significant.

## Results

### TMZ Mitigated Renal Dysfunction in STZ-Induced Diabetic Rats

**Figure 1:**
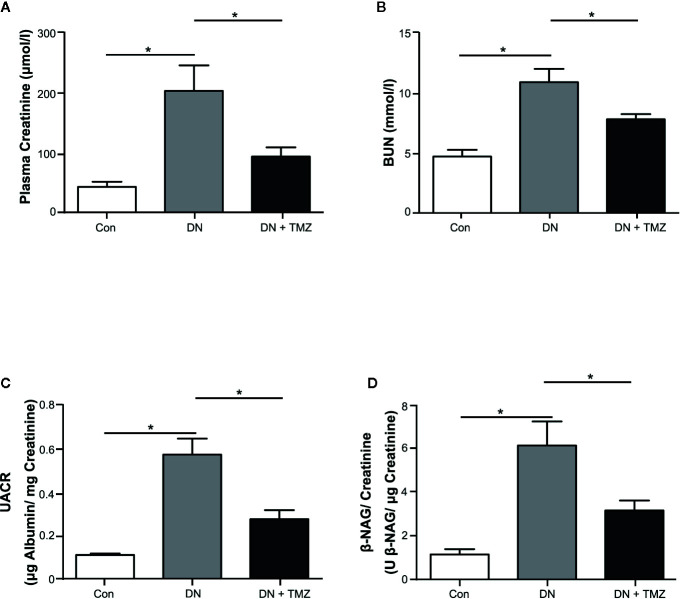
Trimetazidine (TMZ) preserved renal function in streptozotocin (STZ)-induced diabetic rats. Plasma and urine were collected before the experimental rats sacrificed. **(A, B)** Plasma creatinine and BUN determined after 20 weeks of TMZ treatment. **(C, D)** Calculated values of UACR and β-NAG/creatinine in urine samples. BUN, blood urea nitrogen; UACR, urine albumin to urine creatinine ratio; β-NAG, N-acetyl-β-D-glucosaminidase; Con, control group; DN, diabetic nephropathy group; DN + TMZ, TMZ-treated DN group. Data in **(A–D)** were expressed as mean ± SEM (n = 7–8). *P < 0.05 (one-way ANOVA with Newman-Keuls post analysis).

After 20 weeks of STZ injection, metabolic indices associated with diabetes were measured. The weight gain of these rats was significantly reduced, but blood glucose, serum triglyceride and cholesterol levels were markedly increased. Treatment with TMZ in diabetic rats did not change these metabolic indices or blood pressure (**Table 1**). All renal function parameters measured in the study, including plasma creatinine, blood urea nitrogen, urine albumin to urine creatinine ratio, and urine β-NAG to urine creatinine ratio were significantly increased in diabetic rats compared with normal control rats. Interestingly, treating with TMZ blunted these changes markedly (**Figures 1A–D**).

**Table 1:** Biochemical and physical parameters of diabetic rats after TMZ treatment.

Group	Control	DN	DN + TMZ
N	7	8	7
Body weight (g)	566.57 ± 12.30	296.00 ± 15.69*	296.57 ± 9.42
Plasma glucose (mM)	5.84 ± 0.21	26.50 ± 2.56*	26.04 ± 2.81
Plasma triglyceride (mM)	1.29 ± 0.13	3.70 ± 1.02*	3.21 ± 0.79
Plasma cholesterol (mM)	2.02 ± 0.24	4.18 ± 0.41*	4.40 ± 0.44
SBP (mmHg)	141.29 ± 3.65	144.00 ± 3.28	140.60 ± 6.32
DBP (mmHg)	105.67 ± 2.32	95.96 ± 4.38	99.33 ± 4.98

SBP, systolic blood pressure; DBP, diastolic blood pressure.

Values represent mean ± SEM. *P < 0.05 (one-way ANOVA with Newman-Keuls post analysis).

These results indicate that diabetic rats induced by STZ injection experience a remarkable decline in renal function, which can be mitigated by treating with TMZ independent of affecting blood pressure and systemic metabolic levels.

### TMZ Attenuated Glomerular and Tubulointerstitial Remodeling in STZ-Induced Diabetic Rats

**Figure 2:**
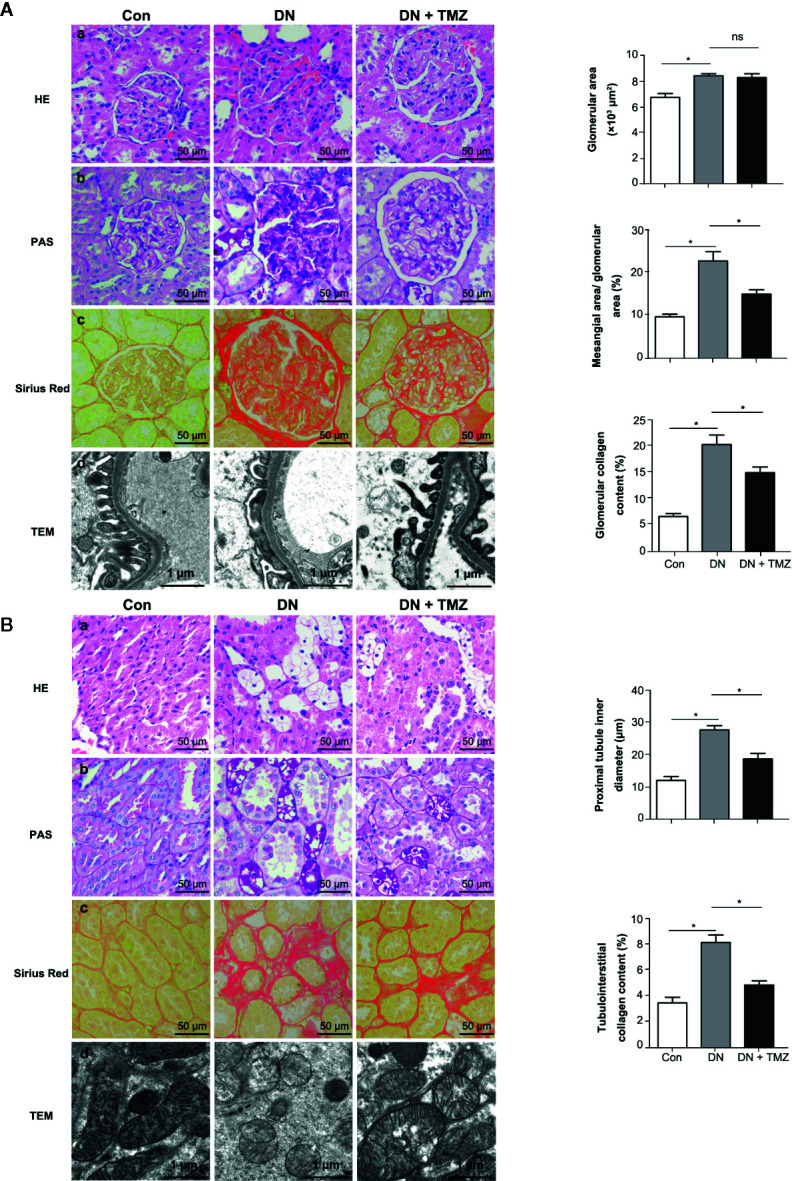
Trimetazidine (TMZ) attenuated glomerular and tubular interstitial remodeling in streptozotocin (STZ)-induced diabetic rats. **(A)** Pathological examination of glomeruli. (a–c) Representative images of HE, PAS, and Sirius Red staining of glomeruli from different groups (left panel). Scale bar, 50 μm. Bar graphs representing analyzed glomerular area, the ratio of mesangial area to glomerular area, glomerular collagen content percentage (right panel). Data were expressed as mean ± SEM (n = 5, the value of each variable was derived from the average of 20 glomeruli per rat). *P < 0.05 (one-way ANOVA with Newman-Keuls post analysis). (d) Representative images of TEM of glomeruli from different groups. Scale bar, 1 μm. **(B)** Pathological detection of tubulointerstitium. (a–c) Representative images of HE, PAS and Sirius Red staining of tubulointerstitium from different groups. Scale bar, 50 μm (left panel). Bar graphs representing analyzed proximal tubule inner diameter, tubulointerstitial collagen content percentage. Data were expressed as mean ± SEM (n = 5, the value of each variable was derived from the average of 20 analyses per rat). *P < 0.05 (one-way ANOVA with Newman-Keuls post analysis). (d) Representative images of TEM of renal tubules from different groups. Scale bar, 1 μm. HE, hematoxylin-eosin staining; PAS, periodic acid schiff staining; Con, control group; DN, diabetic nephropathy group; DN + TMZ, TMZ-treated DN group.

To investigate the effects of TMZ on renal pathological changes in STZ-induced diabetic rats, tissue sections of glomeruli and renal tubules were assessed. Glomerular area, ratio of mesangial area to glomerular area, and ratio of glomerular collagen fiber area to glomerular area (glomerular collagen content percentage) were calculated to evaluate glomerular remodeling. In HE-stained tissue sections, the glomerular area was significantly increased in STZ-injected rats compared to control rats, and treating with TMZ had no effect on glomerular enlargement induced by STZ injection (**Figure 2A-a**). Glomerular mesangial expansion was evaluated by PAS staining. Compared with the control group, the ratio of mesangial area to glomerular area increased significantly in diabetic rats, and was ameliorated by treatment of TMZ (**Figure 2A-b**). Sirius Red staining was used to assess the deposition of glomerular collagen fibers. STZ injection significantly increased the glomerular collagen area, while TMZ treatment partially reversed the collagen deposition (**Figure 2A-c**). To observe the changes of glomerular ultrastructure, transmission electron microscopy (TEM) was used in the experiment. In diabetic rats, the shape of podocytes was irregular and flattened. TMZ treatment alleviated these changes (**Figure 2A-d**).

To evaluate pathological changes of the renal tubules, renal tubular vacuolization, proximal tubule inner diameter, renal interstitial collagen deposition, and renal tubule ultrastructure were investigated. Compared with the control rats, a large number of vacuolated TECs were observed in STZ-induced diabetic rats. This morphological alteration in renal tubular cells was ameliorated by the administration of TMZ (**Figure 2B-a**). In addition, there was a notable increase of the inner diameter of the tubules in the kidneys of diabetic rats, while it was apparently reduced following TMZ treatment (**Figure 2B-b**). On the other hand, collagen deposition increased distinctly in the tubulointerstitial of diabetic rats. This change was also significantly ameliorated in the TMZ treated group (**Figure 2B-c**). The mitochondria were elongated and the mitochondrial crest was clear in the normal group. While in the DN group, the mitochondria were small and round, and the mitochondrial crest was unclear. TMZ treatment improved the mitochondrial morphological changes (**Figure 2B-d**).

These data suggest that STZ-induced diabetic rats experience significant pathological alterations on both glomeruli and renal tubules. TMZ treatment can effectively reverse these changes, at least in part. Particularly, the protective effects of TMZ on DN may be more prominent on the renal tubules than glomeruli.

### TMZ Suppressed Renal Tubular EMT

**Figure 3:**
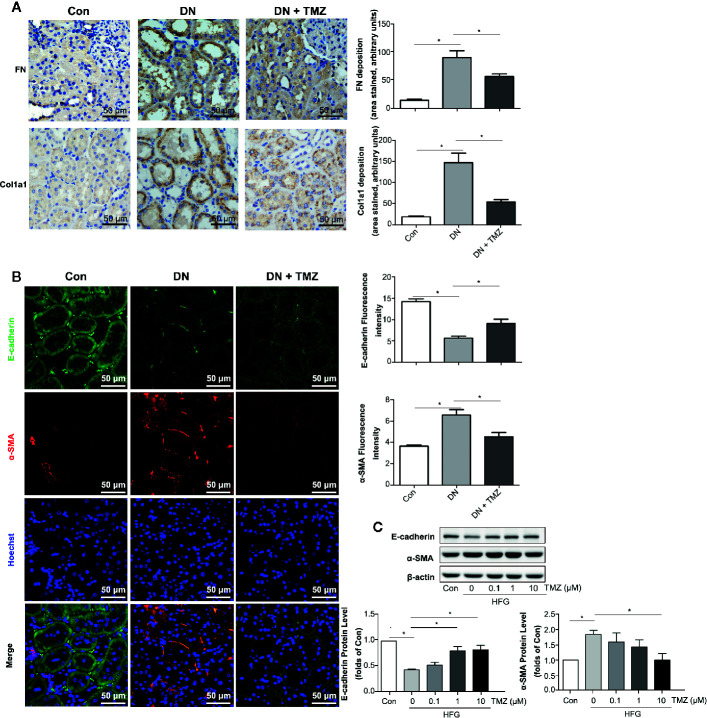
Trimetazidine (TMZ) suppressed renal tubular EMT. **(A)** Relative FN and Col1a1 expression levels in renal cortex measured by immunohistochemical staining. Scale bar, 50 μm. The bar graph illustrated the quantitative analysis of FN and Col1a1 staining. **(B)** Representative images of immunofluorescence staining for E-cadherin (green), α-SMA (red), and Hoechst (blue). Scale bar, 50 μm. The bar graph on the right showed the quantitative analysis of E-cadherin and α-SMA staining. **(A, B)** Con, control group; DN, diabetic nephropathy group; DN + TMZ, TMZ-treated DN group. **(C)** Western blots analysis of the expression of E-cadherin and α-SMA in HK-2 cells treated with different concentrations of TMZ and exposed to HFG for 48 hours. Con, normal control; HFG, high fat and high glucose. Data are expressed as mean ± SEM (n = 3), *P < 0.05 (one-way ANOVA with Newman-Keuls post analysis).

To investigate whether the beneﬁcial effect of TMZ against tubulointerstitial ﬁbrosis was *via* inhibiting EMT in renal tubule, markers of fibrosis and EMT were measured. Immunohistochemical staining of renal tissue sections showed that the expression levels of FN and Col1a1 were predominantly increased in renal tubules of rats that had experienced STZ-injection, while these were markedly decreased after TMZ treatment (**Figure 3A**). Immunofluorescent staining of EMT markers, including E-cadherin and α-SMA, showed that expression of E-cadherin was decreased and expression of α-SMA was increased in renal tubules of rats with STZ-injection, whereas these changes were reversed by TMZ treatment (**Figure 3B**). *In vitro* experiments, HFG-induced EMT in HK-2 cells, characterized by decreased E-cadherin and elevated α- SMA. However, as the treatment concentration of TMZ increased, these alterations were gradually reversed (**Figure 3C**). Vimentin is another marker of cells of mesenchymal origin; β-catenin and E-cadherin are connected in the cytoplasm, and the reduction of E-cadherin will cause the translocation of β-catenin from the cytoplasm to the nucleus (Simic et al., 2013). Vimentin and β-catenin are two other markers of EMT. In this study, their changes were consistent with E-cadherin and α-SMA (**Supplementary Figure 1**).

These results suggest that EMT in tubulointerstitial fibrosis is involved in STZ-induced DN and that treating with TMZ could attenuate this pathological progression.

### TMZ Suppressed EMT by Reducing ROS Production

**Figure 4:**
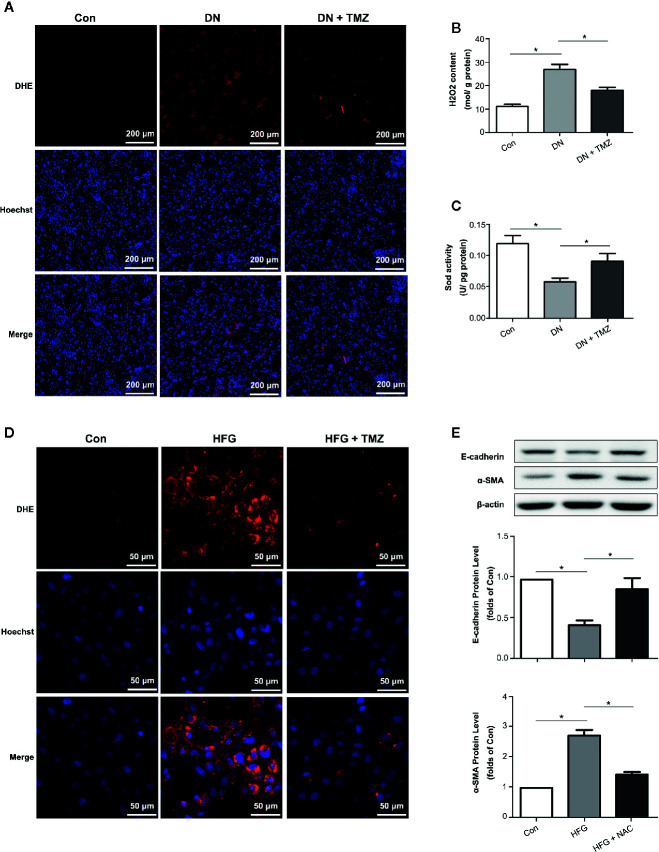
Trimetazidine (TMZ) suppressed HFG-induced EMT by reducing ROS levels in diabetic nephropathy. **(A)** Images of DHE fluorescence in renal cortex from different groups. Scale bar, 200 μm. **(B)** H_2_O_2_ content in renal cortex from different groups. **(C)** Sod activity in renal cortex from different groups. **(A–C)** Con, control group; DN, diabetic nephropathy group; DN + TMZ, TMZ treated DN group. Data were expressed as mean ± SEM (n = 3), *P < 0.05 (one-way ANOVA with Newman-Keuls post analysis). **(D)** Images of DHE fluorescence in HK-2 cells treated with HFG and/or TMZ. Scale bar, 50 μm. **(E)** Western blots analysis of E-cadherin and α-SMA in HK-2 cells treated with HFG and/or NAC. **(B, C)** Con, normal control; HFG, high fat and high glucose; HFG + NAC, high fat and high glucose group treated with NAC. Data were expressed as mean ± SEM (n = 3), *P < 0.05 (one-way ANOVA with Newman-Keuls post analysis).

Oxidative stress has been shown to be associated with EMT. Therefore, DHE staining, H_2_O_2_ level, and Sod activity were performed to investigate the effect of TMZ treatment on ROS production. Results showed that ROS production greatly increased in the kidney tissues of diabetic rats, while TMZ treatment ameliorated the generation of ROS (**Figures 4A–C**). Similarly, TMZ treatment could also inhibit the production of ROS induced by HFG in cultured HK-2 cells (**Figure 4D**). Furthermore, a ROS scavenger called NAC was used to verify whether ROS played a key role in HFG-induced EMT in HK-2 cells. The results showed that NAC could significantly reverse HFG-induced EMT (**Figure 4E**). Therefore, we propose that TMZ may inhibit EMT in DN by reducing ROS levels.

### TMZ Reduced HFG-Induced ROS Generation and EMT Depending on Upregulation of Sirt1

**Figure 5:**
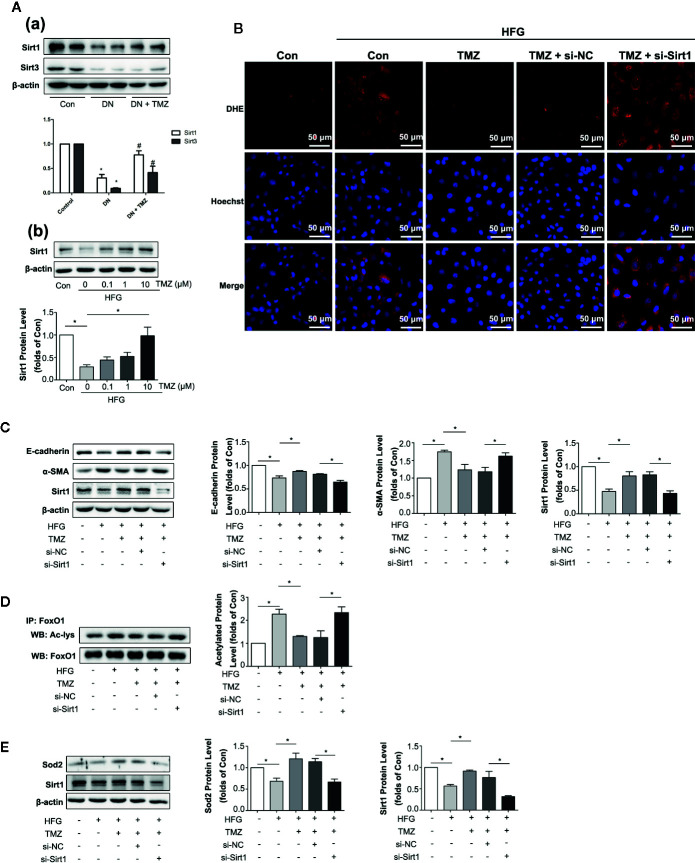
Trimetazidine (TMZ) reduced HFG-induced ROS generation and EMT depending on upregulation of Sirt1. **(A-a)** Western blots analysis of Sirt1 and Sirt3 in the kidneys of diabetic rats and TMZ treated rats. **(A-b)** Western blots analysis of Sirt1 in HK-2 cells treated with HFG and different concentrations of TMZ for 48 h. **(B–E)** HK-2 cells were treated with or without HFG, TMZ, si-NC and si-Sirt1 for 48 h. **(B)** Images of DHE fluorescence. Scale bar, 50 μm. **(C)** Western blots analysis of E-cadherin, α-SMA and Sirt1. **(D)** The relative expression level of acetylated-FoxO1 measured by IP. **(E)** Western blots analysis of Sod2 and Sirt1. Con, normal control; HFG, high fat and high glucose; IP, immunoprecipitation; WB, western blot; Ac-lys, acetyl lysine level. Data were expressed as mean ± SEM (n = 3), *P < 0.05 (one-way ANOVA with Newman-Keuls post analysis).

Previous studies have shown that Sirt1 and Sirt3 have anti-oxidant activity (Wu et al., 2012). We wondered if they were involved in EMT in DN. In addition, they are regulated by the energy state (Noriega et al., 2011), and TMZ is an energy regulator. Therefore, we suspect that TMZ may affect the expression level of them. In our study, both Sirt1 and Sirt3 were significantly down-regulated in DN, and TMZ intervention could significantly restore the expression level of Sirt1. Although the level of Sirt3 was increased in the TMZ treatment group, it was not as obvious as that of Sirt1 (**Figure 5A-a**). Therefore, we mainly study from the perspective of Sirt1. *In vitro* experiments, the Sirt1 protein level was significantly decreased in HK-2 cells when exposed to the HFG environment, and this change was ameliorated by TMZ incubation in a dose-dependent manner (**Figure 5A-b**). DHE fluorescence revealed that the inhibitory effect of TMZ on the ROS production was weakened when Sirt1 was silenced (si-Sirt1) in HFG treated HK-2 cells (**Figure 5B**). After silencing Sirt1, upregulation of E-cadherin and downregulation of α-SMA by TMZ treatment were reversed. This suggested that silencing Sirt1 blunted the role of TMZ in the inhibition of the EMT program induced by HFG (**Figure 5C**). Transcriptional factor FoxO1 is regulated by Sirt1 and plays a role in antioxidative stress (Wu et al., 2012). Therefore, acetylated FoxO1 and the expression level of Sod2, the downstream of antioxidant gene of FoxO1, were examined. While HFG increased the level of acetylated FoxO1, TMZ could inhibit acetylation of FoxO1 and this effect was reversed by silencing Sirt1 (**Figure 5D**). Furthermore, the elevated expression of downstream gene Sod2 was seen by TMZ treatment but diminished after silencing Sirt1 (**Figure 5E**).

These data suggest that HFG induces EMT program in HK-2 cells by enhancing FoxO1 acetylation and reducing expression of its downstream antioxidant proteins, whereas TMZ reverses this process through Sirt1/FoxO1/ROS pathway, specifically by inhibiting the acetylation of FoxO1.

### TMZ Reduced TGFβ1-Induced EMT Depending on Sirt1 Upregulation

**Figure 6:**
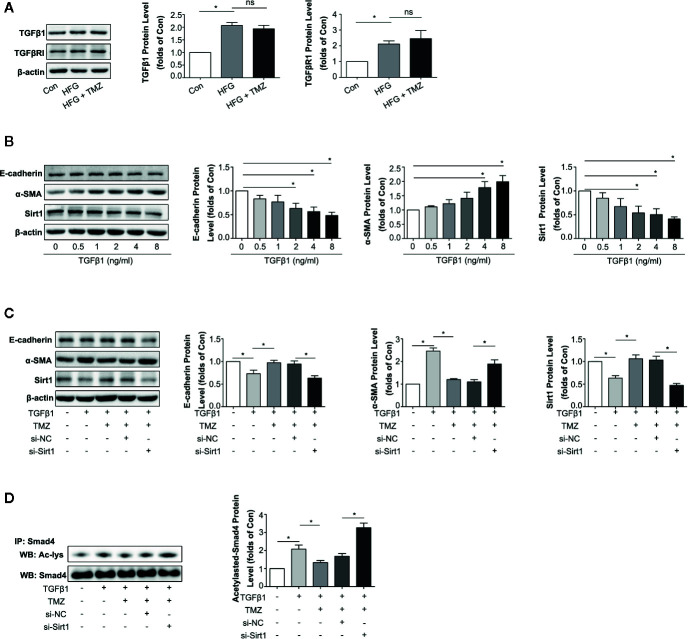
Trimetazidine (TMZ) reduced TGFβ1 induced EMT by a restoration of Sirt1 expression. **(A)** Western blots analysis of TGFβ1 and TGFβRI in HK-2 cells treated with HFG and/or TMZ. **(B)** Western blots analysis of E-cadherin, α-SMA and Sirt1 in HK-2 cells treated with different concentrations of TGFβ1. **(C, D)** HK-2 cells were treated with or without TGFβ1, TMZ, si-NC and si-Sirt1 for 48 h. **(C)** Western blots analysis of E-cadherin, α-SMA and Sirt1. **(D)** The relative expression level of acetylated-Smad4 measured by IP. Con, normal control; IP, immunoprecipitation; WB, western blot; Ac-lys, acetyl lysine level. Data were expressed as mean ± SEM (n ≥ 3), *P < 0.05 (one-way ANOVA with Newman-Keuls post analysis).

TGFβ1 serves as one of the most important cytokines in the process of EMT in kidney (Xu et al., 2009; Iwano, 2010). We then investigated whether TMZ played the role in inhibition EMT through Sirt1/TGFβ1 pathway. We first assessed the expression levels of TGFβ1 and TGFβRI in HK-2 cells after exposure to HFG. The results showed HFG could apparently increase the expression level of TGFβ1 and TGFβRI. However, TMZ had no effect on the elevation of TGFβ1 and TGFβRI induced by HFG (**Figure 6A**). TGFβ1 could induce EMT in HK-2 cells, verified by the decreased expression of E-cadherin and the increased expression level of α-SMA that was seen in a dose dependent manner. These results were accompanied with downregulation of Sirt1 protein levels (**Figure 6B**). Although TMZ had no effect on expression of TGFβ1 and TGFβRI, TMZ intervention could alleviate TGFβ1-induced EMT verified by corresponding changes of E-cadherin and α-SMA expression. Moreover, the effect of TMZ on TGFβ1-induced EMT declined after silencing Sirt1. It suggested that TMZ affected TGFβ1-induced EMT *via* upregulating Sirt1 (**Figure 6C**). Further experiments found TGFβ1 could increase the level of acetylated Smad4, which was reversed after TMZ treatment. However, the effect of TMZ on acetylation of Smad4 was declined after silencing Sirt1 (**Figure 6D**).

These results indicate that HFG can induce EMT *via* the TGFβ1 pathway. TMZ doesn’t affect TGFβ1 or TGFβRI but rather through deacetylation of Smad4 in a Sirt1 dependent way, thereby attenuating TGFβ1-induced EMT.

### TMZ Upregulated Sirt1 Expression in a Nampt/NAD^+^ Dependent Manner in DN

**Figure 7:**
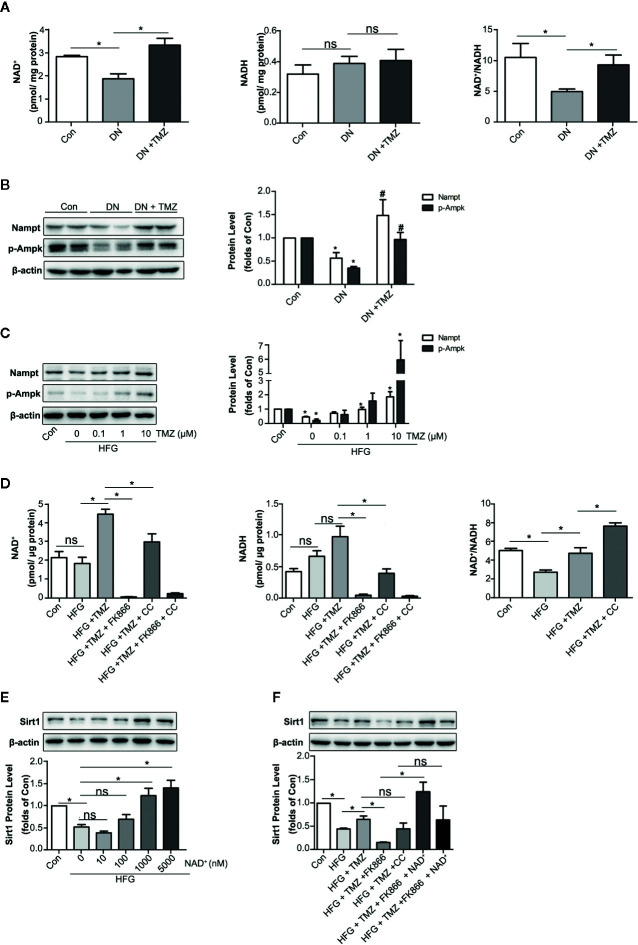
Trimetazidine (TMZ) upregulated Sirt1 expression depending on Nampt/NAD^+^ pathway in diabetic nephropathy. **(A)** The content of NAD^+^ and NADH in TMZ treated DN, and the ratio of NAD^+^ and NADH. **(B)** Western blots analysis of Nampt and p-Ampk in TMZ treated DN. **(C)** Western blots analysis of Nampt and p-Ampk in HK-2 cells treated with different concentrations of TMZ and exposed to HFG for 48 hours. **(D)** The content of NAD^+^ and NADH in HK-2 cells with different treatment, and the ratio of NAD^+^ and NADH. HK-2 cells were treated with 10 μM TMZ and exposed to HFG for 48 h. Nampt activity and Ampk activity were inhibited by FK866 and CC, respectively. **(E)** Western blots analysis of Sirt1 in HK-2 cells treated with different concentrations of NAD^+^ and exposed to HFG for 48 hours. **(F)** Western blots analysis of Sirt1 in HK-2 cells with different treatment. CC, Compound c. Data were expressed as mean ± SEM (n ≥ 3), *P < 0.05 (one-way ANOVA with Newman-Keuls post analysis).

Nicotinamide adenine dinucleotide (NAD^+^) is known for as an essential coenzyme that mediates redox reactions. In addition, it is a substrate for NAD^+^-dependent enzyme such as sirtuins and PARPs (Garten et al., 2015). It has been reported that NAD^+^ affects not only Sirt1 activity, but also its expression level (Revollo and Li, 2013). NADH is the reduced state of NAD^+^, which has the opposite effect on Sirt1 expression (Hayashida et al., 2010). As a metabolic regulator, TMZ may regulate intracellular NAD^+^ level by affecting cellular redox status. In this study, we detected the levels of NAD^+^ and NADH in TMZ treated diabetic rats. The results showed that TMZ increased the content of NAD^+^ and the ratio of NAD^+^ to NADH (NAD^+^/NADH) in diabetic rats (**Figure 7A**). There are many factors that can regulate the NAD^+^ content in cells, of which Nampt and p-Ampk are two important ones (Belenky et al., 2007; Canto et al., 2009). Therefore, we tested their expression. The results showed that TMZ treatment increased the expression of both (**Figures 7B, C**). Inhibiting their activity could significantly reduce the content of NAD^+^, and FK866 (an inhibitor of Nampt) was more effective than Compound C (CC, an inhibitor of Ampk) (**Figure 7D**). Administration of different concentrations of NAD^+^ in HFG-treated HK-2 cells could increase the expression of Sirt1 in a concentration-dependent manner (**Figure 7E**). After the administration of Nampt and Ampk inhibitor, respectively, the up-regulation effect of TMZ on Sirt1 was weakened by FK866 rather than CC (**Figure 7F**). A possible explanation is that CC raised NAD^+^/NADH (**Figure 7D**). Correspondingly, NAD^+^ supplementation rescued the inhibitory effect of FK866 on Sirt1 expression (**Figure 7F**).

These results indicated that regulation of TMZ on Sirt1 expression is dependent on the Nampt/NAD^+^ pathway.

## Discussion

**Figure 8:**
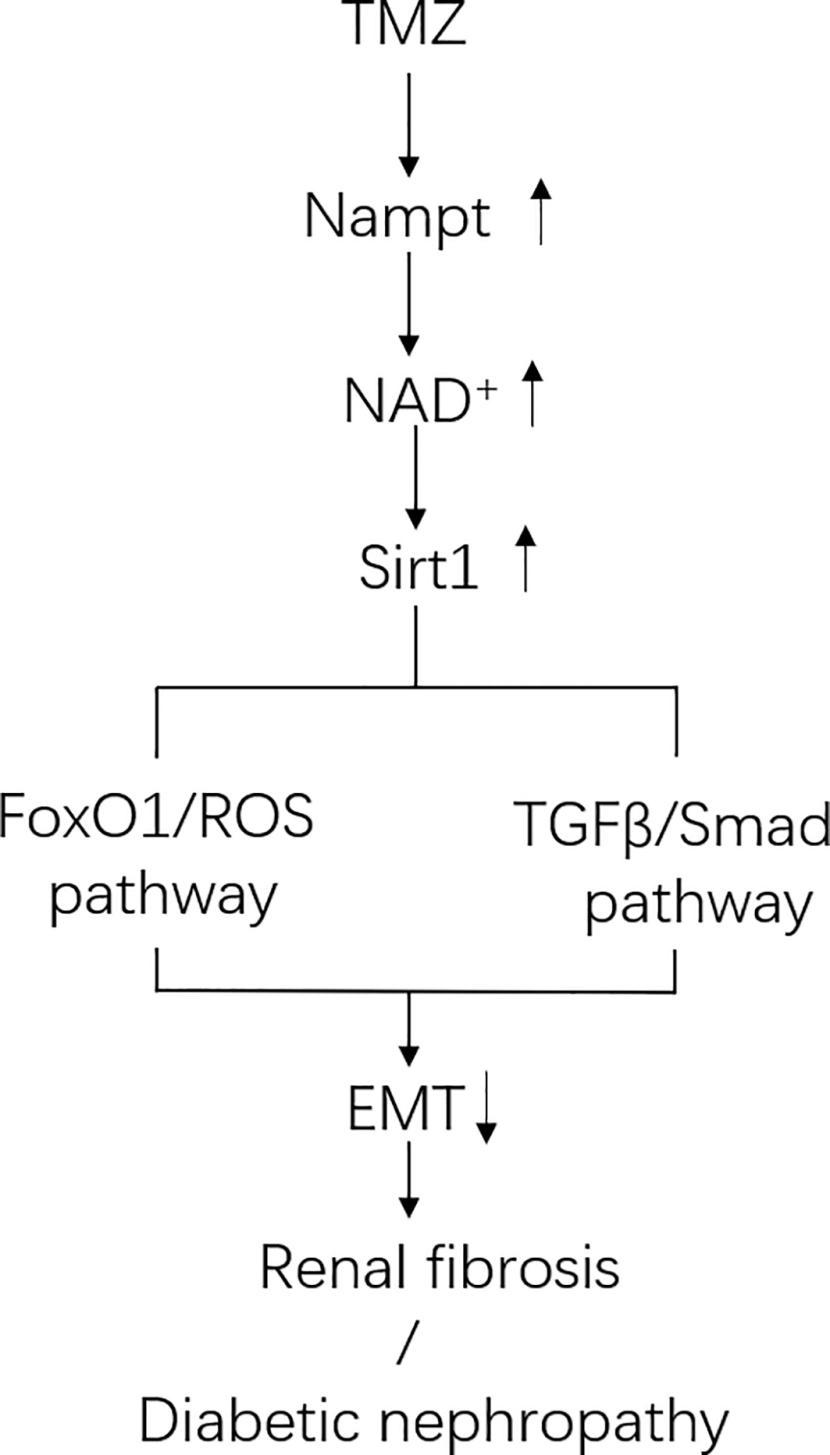
Schematic of pathways involved in Trimetazidine (TMZ)-mediated EMT inhibition in DN. In diabetic environment, ROS pathway and TGFβ/Smad pathway together induce tubule epithelial cells (TECs) to produce EMT. TMZ inhibits EMT generation by suppressing these two pathways *via* upregulating Sirt1 expression in TECs in DN (Sirt1 inhibits the ROS pathway by deacetylating FoxO1, and inhibits TGFβ/Smad pathway by deacetylating Smad4). Both the expression and activity of Sirt1 are regulated by NAD^+^. The generation of NAD^+^ depends on *de novo* synthesis and salvage pathway, and the latter is its main source. Nampt is the rate-limiting enzyme of the salvage pathway. TMZ promotes NAD^+^ production in DN by up-regulating Nampt expression. In conclusion, TMZ inhibits EMT generation by upregulating Sirt1 expression depending on Nampt/NAD^+^ pathway in DN.

In this study, we aimed to investigate the effects of TMZ on DN, specifically on EMT and renal fibrosis. We found that treatment with TMZ could alleviate renal insufficiency and renal pathological remodeling in rats experiencing DN by STZ. This worked partially by the inhibition of EMT. Sirt1/FoxO1/ROS and Sirt1/TGFβ1/Smad4 pathways were involved in the inhibition of EMT by TMZ treatment, and Nampt/NAD^+^ pathway was related to how TMZ affects Sirt1 expression in DN (**Figure 8**).

TMZ is recognized as an anti-ischemic agent because of its metabolic benefits in treating myocardial ischemia. Studies also suggest the beneficial effect of TMZ on other heart diseases, including ischemia reperfusion injury, hypertrophic cardiomyopathy, diabetic cardiomyopathy, and chronic heart failure (D’Hahan et al., 1997; Zhang et al., 2012; Liu et al., 2016; Zhang et al., 2016). The possible role of TMZ in kidney diseases has been suggested by a few studies, which indicate that TMZ may have protective effects on drug or contrast induced nephrotoxicity (Satyanarayana and Chopra, 2002; Chander et al., 2003; Onbasili et al., 2007) and kidney transplant reperfusion injury (Hauet et al., 2000; Faure et al., 2003). The mechanisms are related to the inhibition of inflammatory reactions, reductions in oxidation stress, anti-apoptosis, and improvements in endothelial dysfunction (Onay-Besikci and Ozkan, 2008; Chrusciel et al., 2014). All of these studies were conducted with a focus on AKI. However, the role of TMZ in CKD has rarely been reported. We speculated that TMZ may also have protective effects on CKD. In this study, we observed that early administration of TMZ could alleviate renal insufficiencies and pathological remodeling in STZ-induced diabetic rats. Interestingly, the beneficial effects of TMZ on DN were more prominent in renal tubules than glomeruli. A previous study by Tugba Karadeniz *et al.* reported similar effects of TMZ on DN as well as the finding that TMZ was more protective of the renal tubular and interstitial region by reducing FN and i-NOS expression (Karadeniz et al., 2014). Different from their work, our study focused on the inhibition of tubulointerstitial fibrosis by TMZ.

Our results indicated that TMZ could inhibit tubulointerstitial fibrosis dramatically in diabetic rats. Renal fibrosis represents the final common pathway of CKD, which is mediated by fibroblast activation and their subsequent transformations into myofibroblasts. This results in excessive secretion of ECM, including FN, Col1a1, and ColIV (Eddy, 2014). Other than residual renal fibroblasts, myofibroblasts originated from TECs *via* a process called as EMT also plays an important role in the deposition of ECM (Stahl and Felsen, 2001; Kalluri and Neilson, 2003). We then specifically examined the role of TMZ on EMT program. Our findings suggest that the administration of TMZ can inhibit EMT in diabetic rats and in cultured cells exposed to HFG. Previous study has shown that ROS makes great contribution to EMT program in DN (Ha and Lee, 2003). Meanwhile, TMZ has been shown to have an evident antioxidant effect by inhibiting NADPH oxidase (Liu et al., 2010) or activating the Nrf2/Ho-1 pathway (Wan et al., 2017). These findings suggest that TMZ may inhibit EMT in DN through its antioxidant effect. In our study, TMZ treatment reduced ROS level in DN (**Figure 4**), and TMZ inhibited HFG-induced ROS and EMT production depending on Sirt1 protein level in HK-2 cells (**Figure 5**). Sirt3 also plays an important role in anti-ROS (Morigi et al., 2018). In our study, although Sirt3 decreased significantly as Sirt1 in DN, it increased slightly after TMZ intervention (**Figure 5A-a**). Therefore, Sirt3 may play a weak antioxidant role here. Mitochondria are the main place where cells produce ROS. Due to abnormal mitochondrial morphology and dysfunction, excessive ROS are produced in DN (Sharma, 2016). In present study, TMZ improved mitochondrial morphological abnormalities in renal tubular cells in DN. PGC1α plays an important role in regulating mitochondrial production and function (Scarpulla, 2008). Our results showed that TMZ reduced the acetylation level of PGC1α (**Supplementary Figure 3**). Therefore, TMZ may partially improve the mitochondrial function through the PGC1α pathway, thereby reducing intracellular ROS level.

Down-regulation of Sirt1 expression in proximal tubules has been found in diabetic rats and over-expressing Sirt1 in proximal tubules could prevent kidney injury (Hasegawa et al., 2013). Moreover, Sirt1 inhibits organ fibrosis (Simic et al., 2013) and suppresses oxidative stress (Wu et al., 2012; Zhu et al., 2019), and is regulated by the energy status of the cells (Noriega et al., 2011). TMZ as a metabolic regulator through inhibiting fatty acid oxidation (Kantor et al., 2000; Lopaschuk et al., 2003), so it may regulate Sirt1 expression. In this study, we examined whether Sirt1 could be the action target of TMZ. First, TMZ administration could reverse the decline of Sirt1 *in vivo* and *in vitro* (**Figure 5A**). Second, the effect of TMZ on inhibiting the ROS production and subsequently EMT program induced by HFG *in vitro* was mediated *via* upregulating Sirt1 (**Figures 5B, C**). Third, TMZ reduced the acetylation level of FoxO1 *via* a restoration of Sirt1 expression and then increased the expression of the downstream antioxidant protein Sod2 (**Figures 5D, E**). Therefore, the Sirt1/FoxO1 pathway was involved in the inhibition of ROS production by TMZ. Forth, Although TMZ treatment did not affect HFG-induced upregulation of TGFβ1 and TGFβRI expression, we found it could relieve TGFβ1-induced EMT by reducing acetylated Smad4 level *via* upregulating Sirt1 (**Figure 6**). Therefore, Sirt1/TGFβ1/Smad4 pathway was also involved in the inhibition of EMT by TMZ. In conclusion, our results suggest that Sirt1 may be the key player for the anti-fibrotic effect of TMZ in DN.

In TGFβ/Smad pathway, TGFβ stimulated receptor complex leads to phosphorylation activation of Smad2 and Smad3. Phosphorylated Smad2 and Smad3 form a complex with Smad4 and enter the nucleus, and act as a transcriptional regulator to promote the expression of pro-fibrotic genes. In contrast, the inhibitory Smad6 and Smad7 suppress Smad2/3 activation (Xu et al., 2009). To date, Smad2/3/4/7 have been reported to be deacetylated by Sirt1 (Kume et al., 2007; Simic et al., 2013; Garcia-Vizcaino et al., 2017; Bugyei-Twum et al., 2018). A previous study showed Sirt1 inhibited TGFβ-induced apoptosis in glomerular mesangial cells *via* Smad7 deacetylation (Kume et al., 2007). There are some reports about the deacetylation of Smad2/3 by Sirt1 (Garcia-Vizcaino et al., 2017; Zhang et al., 2017; Bugyei-Twum et al., 2018). However, the deacetylation of Sirt1 on Smad2 is controversial (Simic et al., 2013; Chen et al., 2014). What is clearer is that Sirt1 can obviously deacetylate Smad4 (Simic et al., 2013; Chen et al., 2014). And Smad4, as a common mediator Smad, is necessary for the translocation of TGFβ/Smad family signals into the nucleus. In this study, in order to illustrate the effect of Sirt1 on TGFβ/Smad pathway in DN, we selected only Smad4 for this study. Other Smads, such as Smad2/3/7 may also be involved in the regulation of EMT by TMZ.

It has been reported that NAD^+^ affects both Sirt1 activity and its expression level (Hayashida et al., 2010; Revollo and Li, 2013). In our study, TMZ increased NAD^+^ content in DN. In order to investigate how TMZ affects NAD^+^ content, we conducted a study on Nampt and p-Ampk. The generation of NAD^+^ depends on *de novo* synthesis and salvage pathway, and the latter is its main source. Nampt is the rate-limiting enzyme of the salvage pathway (Belenky et al., 2007). Previous literature reported that Ampk increased NAD^+^ content by increasing mitochondrial β-oxidation (Canto et al., 2009). In our study, only the effect of Nampt on Sirt1 expression was observed, Probably because Ampk reduced NAD^+^/NADH (**Figure 7D**), or because of TMZ inhibition of β-oxidation. In previous studies, although inhibition of Nampt showed deleterious effects, the expression of Nampt was elevated in DN (Yilmaz et al., 2008; Benito-Martin et al., 2014). However, in our study, the expression of Nampt was decreased in DN (**Figures 7B, C** **)**. This may be due to the decreased expression of Nampt in the later stages of DN. The study by Muraoka et al. explained this phenomenon (Muraoka et al., 2019).

In our study, TMZ showed a deacetylation effect by promoting Sirt1 expression. In order to observe whether the combination of TMZ and Sirt1 agonist has a synergistic effect, we administrated both TMZ and RSV in diabetic rats. The results showed both TMZ and RSV could relieve renal insufficiency, ROS, and EMT. However, the combination of the two showed no obvious synergistic effect (**Supplementary Figure 2**). The possible explanation is that, because they have the same mechanism of action, and the dosage of TMZ and RSV has reached the highest concentration of their effect in our experimental design, therefore, the combination of the two does not see a stronger anti-EMT effect.

Currently, the treatment of DN mainly consists of four parts: glycemic control, blood pressure control, inhibition of the renin-angiotensin system, and cardiovascular risk reduction (Umanath and Lewis, 2018). To date, no therapy specifically targeted at renal fibrosis is known. Although new hypoglycemic drugs with additional mechanisms are being tested for DN, such as SGLT2 inhibitor and GLP-1 activator (Warren et al., 2019), drugs targeting at renal fibrosis are needed to be used as adjuvant treatments to prevent the development and progression of DN. Our study demonstrates the renal protective effect of TMZ in relieving tubulointerstitial fibrosis in diabetic rats. Moreover, TMZ rarely has side effects in clinical practice in the past years. Therefore, TMZ is promising as a new adjuvant therapy for DN in the future. In our study, we found that TMZ was a potential Sirt1 agonist. Sirt1 has a protective effect on multiple organs, especially the heart (Gomes et al., 2019). Most patients with chronic renal failure are accompanied by cardiac insufficiency. Therefore, long-term use of TMZ in patients with DN may benefit from multiple organs and display a better result.

In conclusion, our study provides experimental evidences suggesting that TMZ can attenuate the development and the progression of renal dysfunction in DN. The underlying mechanism comes from TMZ’s inhibition of tubulointerstitial fibrosis by blunting EMT. In detail, TMZ inhibits ROS pathway and TGFβ/Smad pathway to relieve EMT in a diabetic environment by upregulating Sirt1. Further clinical research will be needed to verify if TMZ is an effective adjuvant drug, in combination with hypoglycemic agents and other treatments, against DN.

## Limitations

Our study focused on the effect of TMZ on diabetic renal tubular EMT. We did not examine the possible effect of TMZ on other pathological processes, such as apoptosis and inflammation. We also did not investigate the mechanisms of fibroblasts activation other than EMT. Future studies should consider these aspects in order to better understand the role of TMZ in DN.

## Data Availability Statement

All datasets generated for this study are included in the article/**Supplementary Material**.

## Ethics Statement

The animal study was reviewed and approved by The Animal Research Committee of Tongji Medical College.

## Author Contributions

YY contributed to the design of the study, data collection and drafting of this paper. YoW, ZH and YL participated in performing the study. CC, YaW and DW participated in designed the study. HW contributed to the design, organization of the work and prepared the manuscript.

## Funding

This work was supported by National Natural Science Foundation of China (number: 81570367 and 81900341).

## Conflict of Interest

The authors declare that the research was conducted in the absence of any commercial or financial relationships that could be construed as a potential conflict of interest.
