# Supplementary material for: Trimetazidine Inhibits Renal Tubular Epithelial Cells to Mesenchymal Transition in Diabetic Rats via Upregulation of Sirt1
Source: Front Pharmacol. 2020 Jul 29;11:1136. doi: 10.3389/fphar.2020.01136 (PMC7403491; doi:10.3389/fphar.2020.01136)
Supplement: Supplementary file 1 [file DataSheet_1.docx]

**MATERIALS AND METHODS**

**Reagents**

Antibodies against PGCα (66369-1-lg), β-catenin (51067-2-AP), Vimentin (10366-1-AP) and Histone H3 (17168-1-AP) were purchased from Proteintech Group (Wuhan, China). Antibody against α-tubulin (AC012) was from Abclonal (Cambridge, MA, USA). Rresveratrol (RSV, ST1623) were purchased from [Beyotime Biotechnology](http://www.baidu.com/link?url=0xhLpnT0eaPTx1LV_8bu-9H0GW7Bd7PrXmgMVJ3UUyEh5mRzy5vm50Ms0fvHDq7F) (Shanghai China). Nucleus and cytoplasmic protein extraction kit was from Boster (Wuhan, China).

**Animal experiment**

8 weeks old male Sprague-Dawley rats were purchased from Hubei Research Centre of Laboratory Animals (Wuhan, China). Diabetes was induced by intraperitoneal injection of STZ (55 mg/kg). Diabetic rats were randomly divided into 4 groups, namely diabetic nephropathy group (DN), TMZ treatment group (DN + TMZ), RSV treatment group (DN + RSV), TMZ and RSV treatment group (DN + TMZ + RSV). TMZ (5 mg/kg/day) and RSV (30mg/kg/day) were given to rats in different groups by gavage. Blood, urine, and kidneys are collected for the detection of renal function and oxidative stress indexes and immunofluorescence staining for E-cadherin and α-SMA.

**Nuclear and cytoplasmic protein fractionation**

Cytoplasmic and nuclear protein extraction was performed using a cytoplasmic and nuclear protein extraction kit, according to the manufacturer's instructions. In brief, cells were collected and incubated in cytoplasmic protein extraction reagent A at 4°C for 15 minutes. Then added cytoplasmic protein extraction reagent B, and incubated on ice for 1 minute. Mixed well and centrifuged at 16,000g for 5 minutes. The supernatant was the extracted cytoplasmic protein. Added nucleoprotein extraction reagent to the precipitate, and incubated at 4°C for 40 minutes. During this period, mixed every 10 minutes. Then centrifuged at 16,000g for 5 minutes. The supernatant was the extracted nuclear protein.

**PGC1**α **acetylation assay**

PGC1α acetylation level was measured by immunoprecipitation of collected protein lysate using antibody against PGC1α. Subsequently, acetylated-PGC1α was detected using western blot with anti-pan acetyl lysine antibody. The acetylated-PGC1α protein level was normalized to the PGC1α level.

**
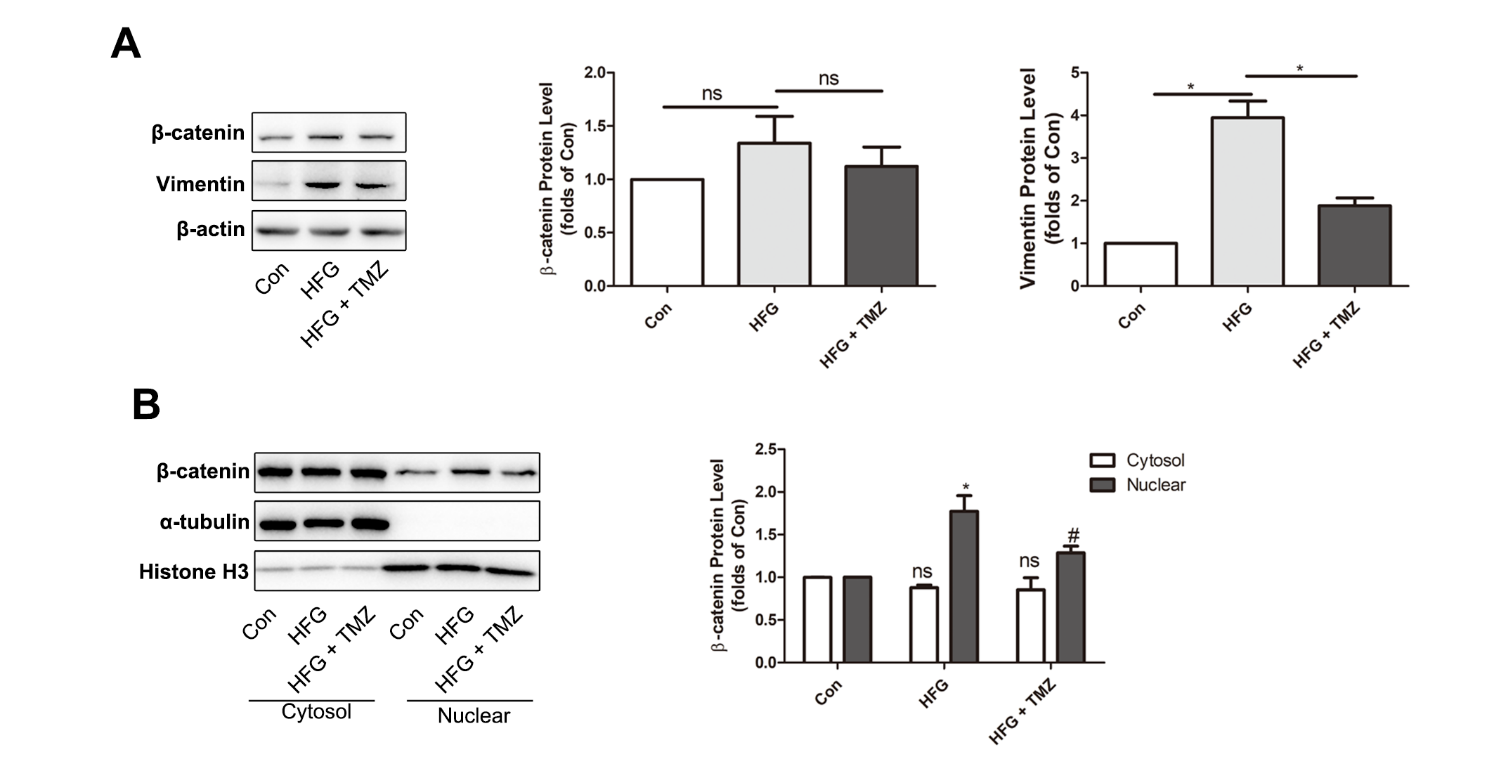
 Supplementary Figure 1 |** TMZ inhibited HFG-induced EMT in HK-2 cells. The translocation of β-catenin from the cytoplasm into the nucleus and the increased expression of Vimentin represent two other markers of EMT in addition to E-cadherin and α-SMA. HK-2 cells were treated with HFG or HFG + TMZ for 48 h. Then collected the cells for analysis. **(A)** Western blots analysis of β-catenin and Vimentin in whole cell lysates. **(B)** Western blots analysis of cytoplasmic and nuclear β-catenin. Data were expressed as mean ± SEM (n ≥ 3), ******P* < 0.05 (One-way ANOVA with Newman-Keuls post analysis).

**
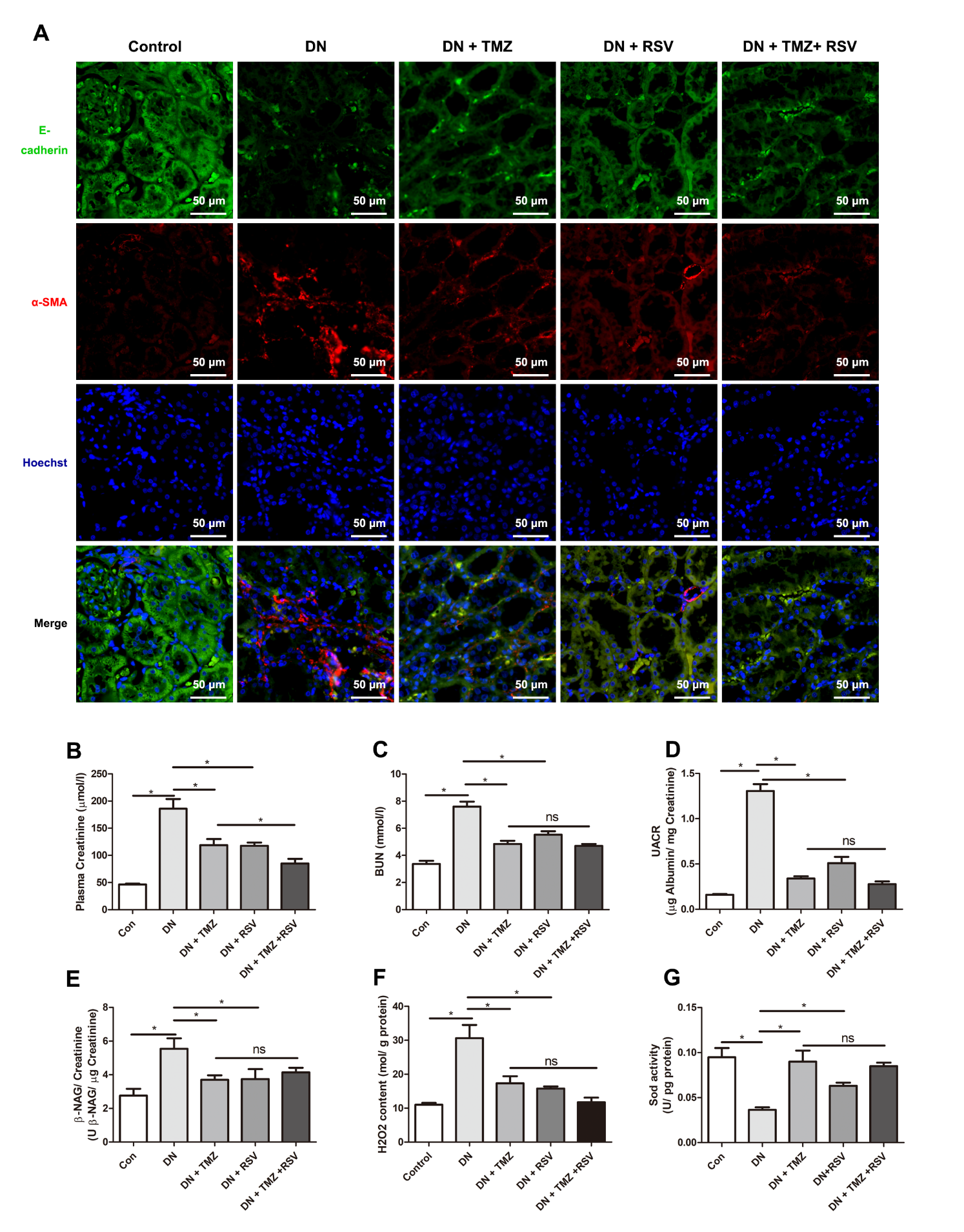
**

**Supplementary Figure 2 |** TMZ and RSV had no synergistic effect against EMT in DN. Diabetic rats were respectively treated with TMZ (5 mg/kg/day), RSV (30 mg/kg/day) and TMZ + RSV by gavage for 8 weeks. Renal cortical tissue was used for fluorescent staining. Blood and urine were used for renal function testing. **(A)** Representative images of immunoﬂuorescence staining for E-cadherin (green), α-SMA (red), and Hoechst (blue). Scale bar, 50 μm. **(B-E)** Renal function indexes. Plasma creatinine, BUN, UACR, and β-NAG/creatinine, respectively. **(F)** H2O2 content in kidney tissues of different treatment groups. **(G)** Sod activity in kidney tissues of different treatment groups. Con, control group; DN, diabetic nephropathy group; DN + TMZ, TMZ-treated DN group; DN +RSV, Rresveratrol treated DN group; DN + TMZ + RSV, TMZ and Rresveratrol treated DN group; BUN, blood urea nitrogen; UACR, urine albumin to urine creatinine ratio; β-NAG, N-acetyl-β-D-glucosaminidase. Data were expressed as mean ± SEM (n ≥ 5), ******P* < 0.05 (One-way ANOVA with Newman-Keuls post analysis).

**
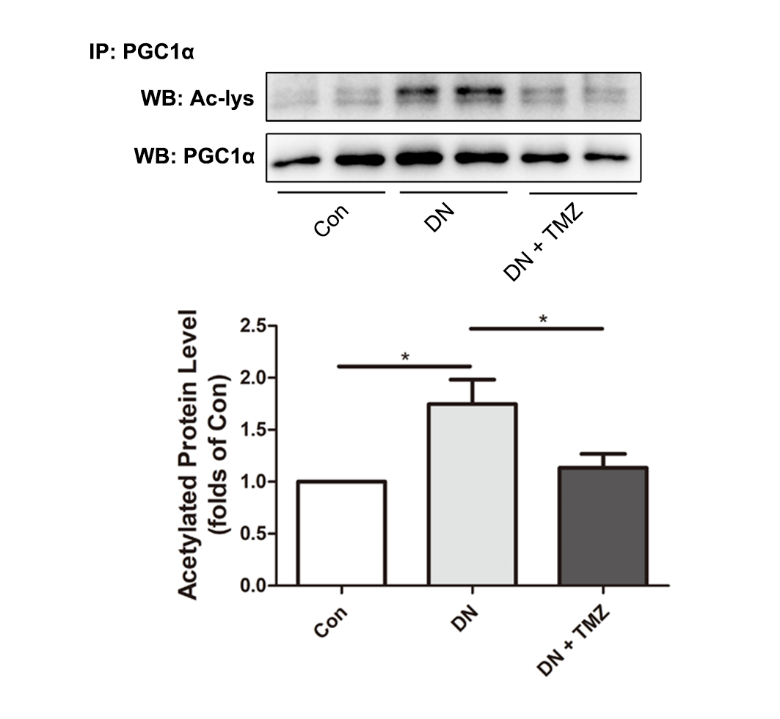
**

**Supplementary Figure 3 |** TMZ reduced PGC1α acetylation level in DN. Western blots analysis of acetylated-PGC1α protein level. Ac-lys, acetyl lysine level. Data were expressed as mean ± SEM (n = 4), ******P* < 0.05 (One-way ANOVA with Newman-Keuls post analysis).
